# *AIP* mutations in Brazilian patients with sporadic pituitary adenomas: a single-center evaluation

**DOI:** 10.1530/EC-17-0237

**Published:** 2017-10-26

**Authors:** Paula Bruna Araujo, Leandro Kasuki, Carlos Henrique de Azeredo Lima, Liana Ogino, Aline H S Camacho, Leila Chimelli, Márta Korbonits, Monica R Gadelha

**Affiliations:** 1 Department of Internal Medicine and Endocrine UnitMedical School and Hospital Universitário Clementino Fraga Filho, Universidade Federal do Rio de Janeiro, Rio de Janeiro, Rio de Janeiro, Brazil; 2 Diagnósticos da América SARio de Janeiro, Rio de Janeiro, Brazil; 3 Neuroendocrinology UnitInstituto Estadual do Cérebro Paulo Niemeyer, Rio de Janeiro, Rio de Janeiro, Brazil; 4 Endocrinology UnitHospital Federal de Bonsucesso, Rio de Janeiro, Rio de Janeiro, Brazil; 5 Molecular Genetics LaboratoryInstituto Estadual do Cérebro Paulo Niemeyer, Rio de Janeiro, Rio de Janeiro, Brazil; 6 Neuropathology Laboratory Instituto Estadual do Cérebro Paulo NiemeyerRio de Janeiro, Rio de Janeiro, Brazil; 7 National Cancer InstituteRio de Janeiro, Rio de Janeiro, Brazil; 8 Centre for EndocrinologyWilliam Harvey Research Institute, Barts and The London School of Medicine, Queen Mary University of London, Charterhouse Square, London, UK

**Keywords:** AIP, germline mutations, sporadic pituitary adenomas, tumor suppressor gene

## Abstract

Aryl hydrocarbon receptor-interacting protein (*AIP*) gene mutations (*AIPmut*) are the most frequent germline mutations found in apparently sporadic pituitary adenomas (SPA). Our aim was to evaluate the frequency of *AIPmut* among young Brazilian patients with SPA. We performed an observational cohort study between 2013 and 2016 in a single referral center. *AIPmut* screening was carried out in 132 SPA patients with macroadenomas diagnosed up to 40 years or in adenomas of any size diagnosed until 18 years of age. Twelve tumor samples were also analyzed. Leukocyte DNA and tumor tissue DNA were sequenced for the entire *AIP*-coding region for evaluation of mutations. Eleven (8.3%) of the 132 patients had *AIPmut*, comprising 9/74 (12%) somatotropinomas, 1/38 (2.6%) prolactinoma, 1/10 (10%) corticotropinoma and no non-functioning adenomas. In pediatric patients (≤18 years), *AIPmut* frequency was 13.3% (2/15). Out of the 5 patients with gigantism, two had *AIPmut*, both truncating mutations. The Y268* mutation was described in Brazilian patients and the K273Rfs*30 mutation is a novel mutation in our patient. No somatic* AIP* mutations were found in the 12 tumor samples. A tumor sample from an acromegaly patient harboring the A299V *AIPmut* showed loss of heterozygosity. In conclusion, *AIPmut* frequency in SPA Brazilian patients is similar to other populations. Our study identified two mutations exclusively found in Brazilians and also shows, for the first time, loss of heterozygosity in tumor DNA from an acromegaly patient harboring the A299V *AIPmut*. Our findings corroborate previous observations that *AIPmut* screening should be performed in young patients with SPA.

## Introduction

Although most pituitary adenomas occur sporadically, with only 5% of all cases being related to inherited syndromes (1), the mechanisms underlying pituitary tumorigenesis in a non-familial setting are poorly understood. Somatic mutations and other genetic and/or epigenetic abnormalities have been related to SPA, but a minor subgroup of these adenomas can have a germline mutation in a predisposing gene with no known familial history of pituitary adenoma (2). Germline aryl hydrocarbon receptor-interacting protein (*AIP*) gene mutations (*AIPmut*) were first described by Vierimaa and coworkers in 2006 (3). This study has found *AIPmut* in seemingly sporadic acromegaly patients and in familial isolated pituitary adenomas (FIPA) (3), which is characterized by the presence of pituitary adenomas in two or more members of the same family in the absence of other syndromic clinical features.

*AIP* appears to act as a tumor suppressor gene (TSG) (3). It is a cytoplasmic protein and a co-chaperone of heat-shock protein 90 (HSP90), and several studies demonstrated the involvement of AIP in various nuclear receptor signaling pathways, such as in estrogen receptor α (ERA) and glucocorticoid receptor (GR) signaling pathways (4, 5, 6). However, the exact molecular mechanisms by which *AIPmut* promotes pituitary adenomas are unclear. There is evidence that a failure to inhibit cyclic adenosine monophosphate (cAMP) synthesis underlies the development of pituitary adenomas in *AIPmut* patients (7). The observation of loss of heterozygosity (LOH) at the chromosome 11q13 in pituitary adenomas containing *AIPmut* provides another argument for the role of these genetic mutations in pituitary tumorigenesis (3, 8). Functional evaluation of *AIPmut* has shown reduced ability to inhibit cell proliferation and disruption of the protein–protein interaction between AIP and phosphodiesterase-4A5 (PDE4A5) (9). In addition, the observation that most pathological mutations lead to a truncated protein, mostly affecting its C-terminal part, which is involved in interactions with other proteins, or conformational changes that lead to altered protein stability (10), reinforces the role of *AIP* as a TSG (11).

A number of studies have investigated the prevalence and the clinical characteristics of patients with all types of apparently SPA and *AIPmut* (12, 13, 14, 15, 16), and so far, it is established that *AIPmut* are the most frequent germline mutations found in SPA (17). The seemingly low prevalence of *AIPmut* in apparently sporadic cases is probably due to low penetrance (20%) (14, 18), as de novo mutations have only been described in 2 patients (19, 20). Patients harboring *AIPmut* are predominantly male (61%), are young at the time of diagnosis (78% aged <30 years) and tend to have macroadenomas (88%) with extrasellar extension making curative surgery less likely (21). In case of acromegaly, *AIPmut* patients have a poor response to medical treatment (14, 21). Therefore, recognition of *AIPmut* positive pituitary adenomas is of clinical importance and family member screening can provide early diagnosis of affected patients not yet diagnosed leading to higher chance of disease control.

The findings of previous studies that investigated the prevalence of germline *AIPmut* in patients with SPA suggest that screening should be focused on young patients (diagnosed before the age of 30–40 years) with macroadenomas or in patients with any size of tumors diagnosed before age 18 years (22). The studies that applied those criteria have found a prevalence ranging from 2.8 to 11.7% (13, 14, 16, 23, 24, 25). Most studies were performed in European populations, and only a few were multicentric (14, 16, 26). Although prevalence of *AIPmut* seems to be similar across different ethnicities, new studies can show variations in *AIPmut* profiles and bring more data from different populations. Thus, we analyzed patients with SPA, with diagnosis up to 40 years, for the presence of *AIPmut* in our tertiary referral center in Brazil.

## Materials and methods

### Subjects

Consecutive patients with SPA from a single referral center were prospectively enrolled from July 2013 to February 2016. This tertiary referral center is part of a University Hospital established in Rio de Janeiro, which is linked to the single health system of Brazil, receiving referrals from all the State. Inclusion criteria were evidence of macroadenoma (maximal diameter ≥10 mm on pituitary MRI) diagnosed up to 40 years. Patients with diagnosis until 18 years of age (pediatric patients) were included both with micro or macroadenoma. Clinical, laboratory and family history from all subjects was undertaken to exclude familial pituitary adenomas either isolated (FIPA and X-linked acro gigantism) or as a component of other genetic syndromes (e.g. multiple endocrine neoplasia types 1 (MEN1) and 4 (MEN4), Carney complex, familial pheochromocytoma/paraganglioma/pituitary adenoma syndrome) (27). Genomic analyses for the screening of these genetic syndromes were not performed. All subjects gave written informed consent. The Ethics Committee of the Medical School and the Hospital Universitário Clementino Fraga Filho of the Universidade Federal do Rio de Janeiro (HUCFF-UFRJ) approved the study. Genetic counseling was provided for family members of *AIPmut*-positive cases, and clinical testing and follow-up were offered, whenever possible.

### Pituitary tumor samples

Formalin-fixed and paraffin-embedded tissue was available from patients who underwent surgery in our center. Histological sections were stained with H&E and submitted to immunohistochemical reactions for pituitary hormones (GH – dilution 1:5000, PRL – dilution 1:5000, ACTH – dilution 1:4000, FSH – dilution 1:3000, TSH – dilution 1:2000, LH – dilution 1:4000), all of them polyclonal rabbit antibody/cell marque. In addition, GH-positive tumors were immunostained with CAM 5.2 (monoclonal mouse antibody cytokeratin (CAM5.2)/Cell Marque, dilution 1:2000) to differentiate sparsely from densely granulated tumors.

Nine frozen and three paraffin-embedded tissue samples of the enrolled patients were available for genetic screening for somatic *AIP* gene mutations. In cases where *AIPmut* were identified in leukocyte DNA and tumor DNA was available, search for LOH was performed through *AIP* sequencing.

### Genomic analyses of *AIP*

Mutation screening of *AIP* was done using genomic DNA isolated from peripheral blood leukocytes and from frozen or paraffin-embedded tumor tissues, using the Gentra PureGene Blood Kit (Qiagen), AllPrep DNA/RNA/miRNA Universal Kit (Qiagen) and QIAamp DNA FFPE Tissue Kit (Qiagen), respectively, following the manufacturer’s instructions.

The entire *AIP*-coding region (exons 1–6) as well as flanking intronic sequences were amplified and sequenced with *AIP* PCR/sanger sequencing primer pairs (Thermo Fisher Scientific). The promoter region was not analyzed. PCRs were performed on Applied Biosystems ProFlex PCR System (Applied Biosystems). PCR products clean up were performed with ExoSAP-IT (USB Corporation, Cleveland, OH, USA). DNA sequencing was performed using Big Dye Terminator 3.1 Cycle Sequencing kit and an automated capillary sequencer (ABI 3130xl Genetic Analyzer, Applied Biosystems). Electropherogram-derived sequences were compared with NCBI references for the *AIP* gene (NG_008969.1 RefSeq-Gene and NM_003977.3 transcript) using Benchling (http://benchling.com, Benchling Inc, San Francisco, CA, USA). All genetic alterations were confirmed by a repeated analysis.

*AIP* sequence variants were compared with human single-nucleotide polymorphism (SNP) databases (dbSNP, http://www.ncbi.nlm.nih.gov/SNP/snp_summary.cgi), ExAC database (http://exac.broadinstitute.org) and also against *AIP* mutation data from genetically diverse populations (28). Only the variants that met the mutation criterion, defined as a minor allele frequencies (MAF) <1%, were considered for further analysis (intronic variants outside the splicing site area were not analyzed). PolyPhen2 (http://genetics.bwh.harvard.edu) and Alamut Software, version 2.2e (Interactive Biosoftware, Rouen, France) were used to evaluate the pathogenicity of missense mutations on AIP structure. Mutations were classified as pathogenic, likely pathogenic, variants of uncertain significance (VUS), likely benign or benign, according to the Standards and Guidelines for the Interpretation of Sequence Variants (29).

Patients with somatotropinomas and pediatric patients with any pituitary adenoma in whom *AIP* sequencing did not find a mutation were screened for large deletions of the *AIP* using multiplex ligation-dependent probe amplification (Salsa MLPA probemix P244-B1 AIP-MEN1, MRC-Holland, Amsterdam, The Netherlands), whenever suitable quality DNA was available.

### Statistical analyses

Normal distribution was tested by the Kolmogorov–Smirnov and Shapiro–Wilk tests. The Mann–Whitney *U* test and the *χ* ^2^ test were used for statistical analysis. Data are given as median (range). *P* values below 0.05 were considered as significant.

## Results

### Clinical characteristics of the study cohort

A total of 132 patients with sporadic pituitary macroadenomas diagnosed up to 40 years, and with micro or macroadenomas diagnosed until 18 years of age were included. Of these patients, 74 (56%) had acromegaly or gigantism, 38 (28.8%) had prolactinoma, 10 (7.6%) had non-functioning pituitary adenoma (NFPA) and 10 (7.6%) had Cushing’s disease. The median age at diagnosis was 28 (9–40) years, 15 (11.3%) had diagnosis during childhood or adolescence (age ≤18 years), 84 (63.6%) were female and the median tumor diameter at diagnosis was 22 (6–81) mm. Characteristics of each group at diagnosis are given in Table 1. Female predominance was seen in all groups.

**Table 1:** Clinical, radiological and pathological data of the study cohort.

**Type of pituitary tumor and distribution** (Dx until and after 18 years)	**Females** (%)	**Median age** (min–max)	**Median tumor diameter mm** (min–max)	**Giant adenomas** (%)	**Available tumors**
Somatotropinoma	43 (58)	29 (18–40)	24 (11–61)	7 (9.4)	8
*n* = 74; 56.0%					
≤18 years = 1	1 (100)	18	20	0	0
>18 years = 73	42 (58)	29 (20–40)	25 (11–61)	7	8
Prolactinoma	27 (71)	23 (11–40)	19 (10–81)	12 (31.6)	2
*n* = 38; 28.8%					
≤18 years = 8	5 (63)	17 (11–18)	19 (14–60)	3	0
>18 years = 30	22 (73)	25 (19–40)	19.5 (10–81)	9	2
NFPA	6 (60)	30.5 (11–37)	27 (12–50)	1 (10)	0
*n* = 10; 7.6%					
≤18 years = 2	0	12.5 (11–14)	22*	0	0
>18 years = 8	6 (75)	32.5 (22–37)	28.5 (12–50)	1	0
Cushing’s disease	8 (80)	21.5 (9–39)	12 (6–35)	0	2
*n* = 10; 7.6%					
≤18 years = 4	2 (50)	14 (9–16)	10 (6–11)	0	1
>18 years = 6	6 (100)	25 (21–39)	15.5 (12–35)	0	1
Total	84 (63)	28 (9–40)	22 (6–81)	20 (14.5)	12
*n* = 132					

*Only one patient had tumor diameter available.

Dx, diagnosis; NFPA, non-functioning pituitary adenoma.

### Patients with *AIPmut*

Germline *AIPmut* were observed in 11 (8.3%) of the 132 patients. Among these 11 patients, we found 8 different *AIPmut* (3 pathogenic mutations, 3 VUS and 2 likely benign mutations) (Table 2).

**Table 2:** Clinical, radiological and genetic characteristics of patients with *AIP* germline mutations.

**Dx**	**Sex**	**Age at Dx** (years)	**Adenoma size at Dx** (mm)	**Mut**	**Protein change**	**Protein location**	**Functional study**	**Sig**	**PolyPhen2** (*in silico*)	**Alamut** (*in silico*) (SIFT/Mut Taster)	**MAF**	**Type of mut**	**dbSNP reference**
Acro	M	33	12	c.47G > A	R16H	N-terminal	Incomplete loss of interaction with PDE4A5 (11, 35)Similar half-life to WT AIP (10)	Likely benign	Probably damaging (0.966)	Deleterious (0.01)/disease causing (p.0.9)	0.0020	Mis	rs145047094
Acro	F	34	25	c.382C > T	R128C	Between FKBP PPIase and TPR1 domains	NA	VUS	Benign (0.228)	Deleterious (0.04)/polymorphism (p.1.0)	0.0001	Mis	rs140530307
Acro	M	34	Macro	c.382C > T	R128C	Between FKBP PPIase and TPR1 domains	NA	VUS	Benign (0.228)	Deleterious (0.04)/polymorphism (p.1.0)	0.0001	Mis	rs140530307
Giant	M	27	17	c.804C > A	Y268*	TPR3 domain/predicted protein lacking the final 63 aa	Rapid degradation of truncated AIP (10)	Pathogenic			NA	Non	rs121908356
Giant*	F	22	22	c.816delC	K273Rfs*30	TPR3 domain	NA	Pathogenic			NA	Frame	In process
Acro	M	40	19	c.896C > T	A299V	C-terminal α-helix	Incomplete loss of interaction with PDE4A5 (11, 35)Short half-life compared to WT AIP (10)	VUS	Possibly damaging (0.934)	Deleterious (0.04)/disease causing (p.0.9)	0.0004	Mis	rs148986773
CD	M	15	10	c.896C > T	A299V	C-terminal α-helix	Incomplete loss of interaction with PDE4A5 (11, 35)Short half-life compared to WT AIP (10)	VUS	Possibly damaging (0.934)	Deleterious (0.04)/disease causing (p.0.9)	0.0004	Mis	rs148986773
PRL	M	18	60	c.911G > A	R304Q	C-terminal α-helix	No significant reduction in β-galactosidase activity for the R304Q AIP mutant (11, 35)Similar half-life to WTAIP (10)	Pathogenic	Benign (0.047)	Deleterious (0.04)/disease causing (p.0.8)	0.0015	Mis	rs104894190
Acro	F	37	31	c.*14C > A		Not in protein (3′UTR)	NA	VUS			0.0005		rs142567224
Acro	F	33	40	c.*14C > A		Not in protein (3′UTR)	NA	VUS			0.0005		rs142567224
Acro	M	38	25	c.*64G > A		Not in protein (3′UTR)	NA	Likely benign			0.0062		rs115346238
Dx	Sex	Age at Dx (years)	Adenoma size at Dx (mm)	Mut	Protein change	Protein location	Functional study	Sig	PolyPhen2 (*in silico*)	Alamut (*in silico*) (SIFT/mut Taster)	MAF	Type of mut	dbSNP reference
Acro	M	33	12	c.47G > A	R16H	N-terminal	Incomplete loss of interaction with PDE4A5 (11, 35)Similar half-life to WT AIP (10)	Likely benign	Probably damaging (0.966)	Deleterious (0.01)/disease causing (p.0.9)	0.0020	Mis	rs145047094
Acro	F	34	25	c.382C > T	R128C	Between FKBP PPIase and TPR1 domains	NA	VUS	Benign (0.228)	Deleterious (0.04)/polymorphism (p.1.0)	0.0001	Mis	rs140530307
Acro	M	34	Macro	c.382C > T	R128C	Between FKBP PPIase and TPR1 domains	NA	VUS	Benign (0.228)	Deleterious (0.04)/polymorphism (p.1.0)	0.0001	Mis	rs140530307
Giant	M	27	17	c.804C > A	Y268*	TPR3 domain/predicted protein lacking the final 63 aa	Rapid degradation of truncated AIP (10)	Pathogenic			NA	Non	rs121908356
Giant*	F	22	22	c.816delC	K273Rfs*30	TPR3 domain	NA	Pathogenic			NA	Frame	In process
Acro	M	40	19	c.896C > T	A299V	C-terminal α-helix	Incomplete loss of interaction with PDE4A5 (11, 35)Short half-life compared to WT AIP (10)	VUS	Possibly damaging (0.934)	Deleterious (0.04)/disease causing (p.0.9)	0.0004	Mis	rs148986773
CD	M	15	10	c.896C > T	A299V	C-terminal α-helix	Incomplete loss of interaction with PDE4A5 (11, 35)Short half-life compared to WT AIP (10)	VUS	Possibly damaging (0.934)	Deleterious (0.04)/disease causing (p.0.9)	0.0004	Mis	rs148986773
PRL	M	18	60	c.911G > A	R304Q	C-terminal α-helix	No significant reduction in β-galactosidase activity for the R304Q AIP mutant (11, 35)Similar half-life to WTAIP (10)	Pathogenic	Benign (0.047)	Deleterious (0.04)/disease causing (p.0.8)	0.0015	Mis	rs104894190
Acro	F	37	31	c.*14C > A		Not in protein (3′UTR)	NA	VUS			0.0005		rs142567224
Acro	F	33	40	c.*14C > A		Not in protein (3′UTR)	NA	VUS			0.0005		rs142567224
Acro	M	38	25	c.*64G > A		Not in protein (3′UTR)	NA	Likelybenig n			0.0062		rs115346238

*Previously reported by Hernandez-Ramirez *et al.* (14).

Aa, amino acid; Acro, acromegaly; CD, Cushing’s disease; Dx, diagnosis; F, female; FKBP PPIase domain, FK506-binding protein peptidyl-prolyl cis-trans isomerase domain; frame, frameshift; giant, gigantism; M, male; Macro, macroadenoma; MAF, minor allele frequency (ExAC database was used for all *AIPmut*, except for the *AIPmut* c.*64G > A, where dbSNP database was used); Mis, missense; Mut, mutation; NA, not available; non, nonsense; PRL, prolactinoma; PDE4A5, phosphodiesterase-4A5; Sig, significance; TPR, tetratricopeptide repeat; VUS, variant of uncertain significance; UTR, untranslated region; WT, wild type.

Among the 74 patients with somatotropinomas, 9 (12.2%) presented *AIPmut*. From this group, 2 out of 5 patients with gigantism had a pathogenic truncating *AIPmut* (Y268* and K273Rfs*30) and 7 out of 69 (10.1%) patients with acromegaly had an *AIP* VUS or likely benign mutations (Table 2). Dosage analysis by MLPA was possible from 59 of the 65 patients with somatotropinomas without *AIPmut*, including the 3 patients with gigantism, and did not reveal any large deletions.

**Figure 1:**
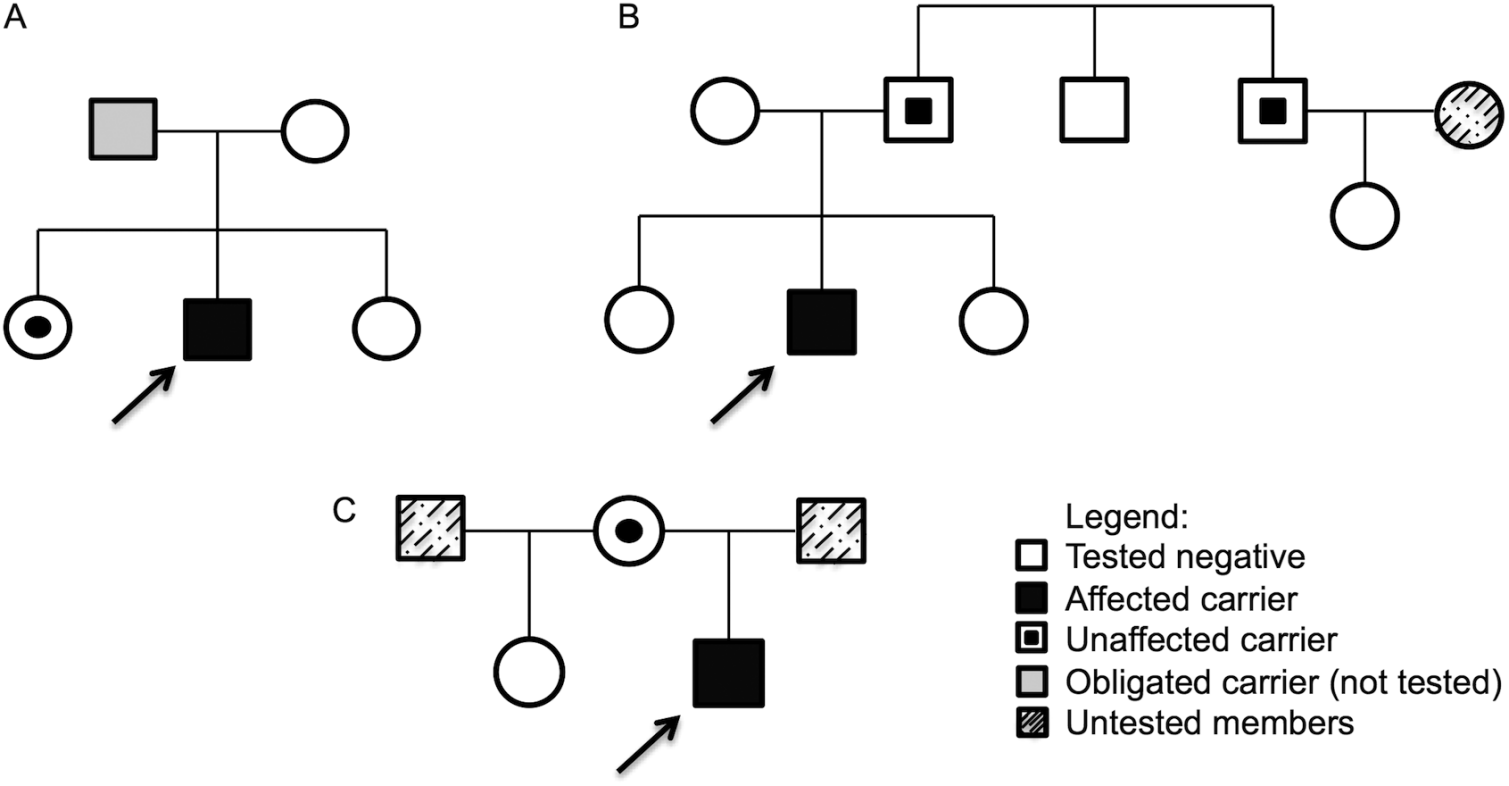
Pedigrees of the families of the probands. The scheme shows the three family trees (A, B and C) of the probands (black squares with an arrow). Male family members are represented by squares, females by circles.

The male patient with gigantism harboring the nonsense *AIPmut* Y268* (c.804C > A), had the diagnosis at 27 years with a history of accelerated growth since the age of 13 years (height at diagnosis 217 cm), arthralgia and a 17 mm macroadenoma. He refused surgery, and treatment with first-generation somatostatin analogue (SA) did not result in normalization of his GH and IGF-1 levels. Parental DNA from his mother and 2 sisters were available for *AIPmut* screening, and the same mutation was found in one of the sisters who is clinically unaffected, although a proper evaluation with pituitary hormones and pituitary MRI was not performed due to her refusal (Fig. 1A).

The frameshifit *AIPmut* K273Rfs*30 (c.816delC) (14) was detected in a 22-year-old female patient who presented with a phenotype of gigantism, height of 181 cm and a 22 mm macroadenoma. She underwent a pituitary surgery, and histopathology of the tumor confirmed to be a somatotropinoma. She was started on clinical treatment with first-generation SA and cabergoline (CAB) with poor response, and then was started on pegvisomant. Unfortunately, genetic screening of her family is not available.

The missense likely benign *AIPmut* R16H (c.47G > A) was found in a male acromegaly patient with diagnosis at the age of 33 years with a macroadenoma of 12 mm. Pituitary surgery was curative and pathology demonstrated a sparsely granulated (SG) somatotropinoma. The other *AIP* likely benign mutation c.*64G > A, located at the 3′ untranslated region (3′UTR), was found in a male acromegaly patient (Table 2), diagnosed at the age of 38 years with a tumor of 25 mm, in a pre-operative evaluation for rhinoplasty. He underwent two pituitary surgeries, and the pathology revealed a SG somatotropinoma. Due to resistance to combined first-generation SA and CAB therapy, the patient was started on pasireotide LAR, which resulted in disease control.

**Figure 2:**
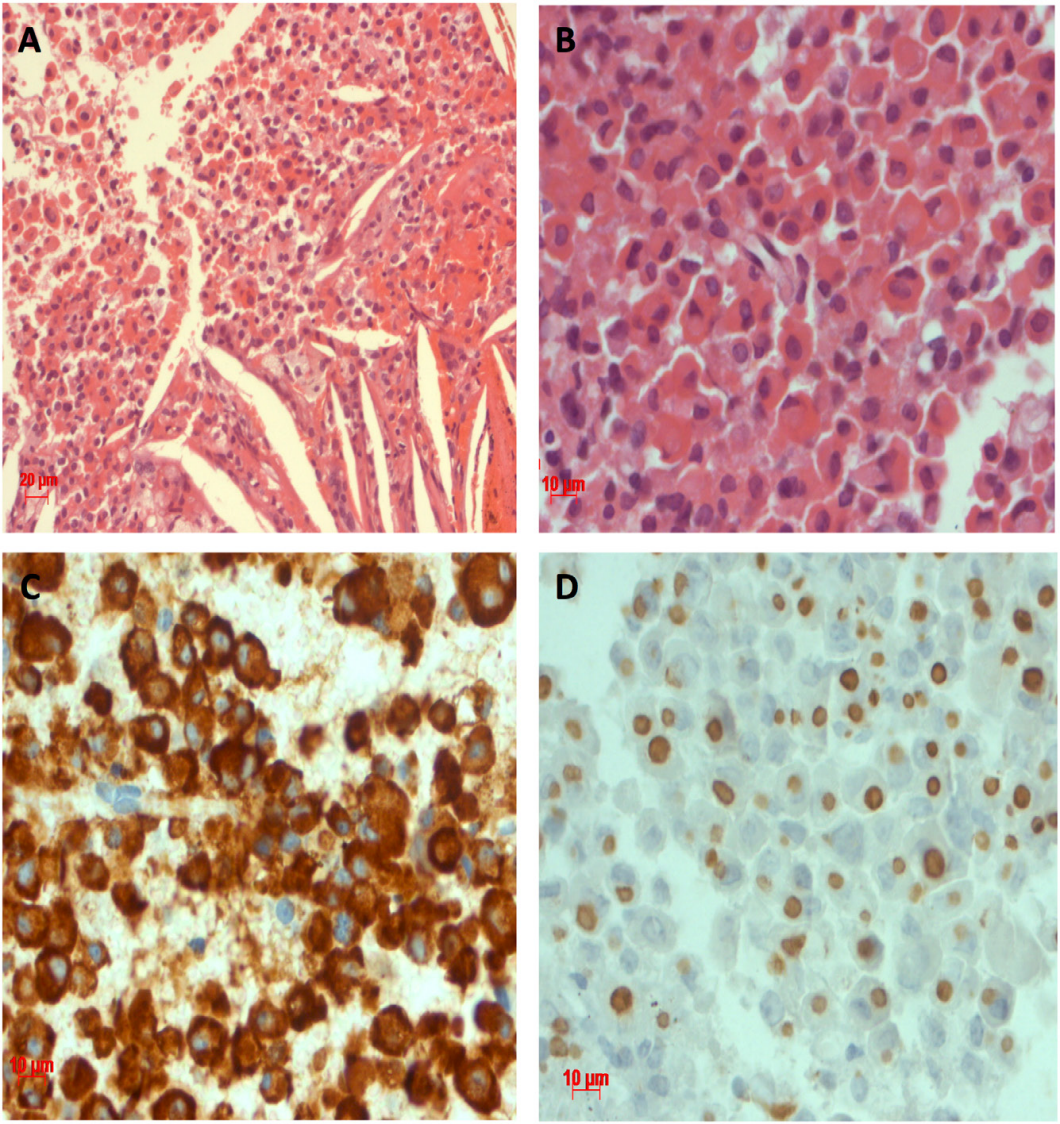
Sparsely granulated somatotropinoma from the patient with the *AIPmut* c.*14C > A. Pituitary adenoma stained with Hematoxilin & Eosin, consisting of eosinophilic cells (A and B) and immunopositive for GH (C), which are sparsely granulated (dot staining) with CAM 5.2 (D). There are blood cells and cholesterol clefts among the epithelial cells (A).

The missense *AIP* VUS A299V (c.896C > T) was present in a male patient with acromegaly diagnosed at the age of 40 years with a tumor of 19 mm. A non-curative pituitary surgery was performed and the pathology showed a SG somatotropinoma. Medical treatment with first-generation SA was started with poor response. The other *AIP* VUS were found only in acromegaly patients, including the missense R128C (c.382C > T) at exon 3 and the 3′UTR c.*14C > A (Table 2). The R128C *AIPmut* was found in one male and one female patient, both diagnosed at the age of 34 years with macroadenomas. Both had non-curative surgeries. The female patient showed resistance to first-generation SA therapy, even after radiation therapy and to CAB association and is now under control with SA and pegvisomant. The male patient had radiotherapy and is under control with first-generation SA treatment. The c.*14C > A *AIPmut* was found in two female patients with acromegaly. The first one was diagnosed at the age of 37 with a tumor of 31 mm operated transsphenoidally. She had resistance to combined treatment of first-generation SA and CAB, but achieved control with the combination of SA and pegvisomant. The second one was diagnosed at the age of 33 years with a giant tumor. She was submitted to a pituitary surgery and the pathology showed a SG somatotropinoma (Fig. 2). Medical treatment with first-generation SA was started, and the patient did not achieve disease control.

In the 38 patients with prolactinomas, 1 (2.6%) had the pathogenic *AIPmut* R304Q (c.911G > A). This male patient was diagnosed in the age of 18 years with a giant adenoma (60 mm) and very high prolactin (PRL) levels (20,000 ng/mL). A pituitary surgery was performed, but PRL remained elevated, and he showed resistance to high doses of CAB (3.5 mg/week). Tumor sample was not available for LOH analysis, but genetic screening of his family identified his father and paternal uncle as *AIPmut* carriers, since clinical evaluation, pituitary hormones dosage and pituitary MRI were normal for both relatives (Fig. 1B).

Among the 10 patients with corticotropinomas, 1 (10%) had the missense *AIP* VUS A299V (c.896C > T). Cushing’s disease was diagnosed in this male patient at the age of 15 years. He had a 10 mm pituitary adenoma that was surgically resected four times, with immunohistochemistry positive for adrenocorticotrophic hormone (ACTH) and a Ki-67 index of 5%. As the patient was not cured, a bilateral adrenalectomy was performed for disease control, and he developed Nelson’s syndrome 6 months after surgery. The same mutation was found in his clinically unaffected mother, who has presented normal levels of pituitary hormones and a normal pituitary MRI (Fig. 1C). This patient’s tissue sample has been tested negative for somatic ubiquitin-specific protease 8 (USP8) gene (30).

No mutations were detected among patients with NFPA. There was no difference regarding age, gender and tumor size at diagnosis between patients harboring or not *AIPmut* (*P* = 0.27, *P* = 0.053 and *P* = 0.94, respectively).

### *AIPmut* in pituitary tumor samples and LOH analysis

**Figure 3:**
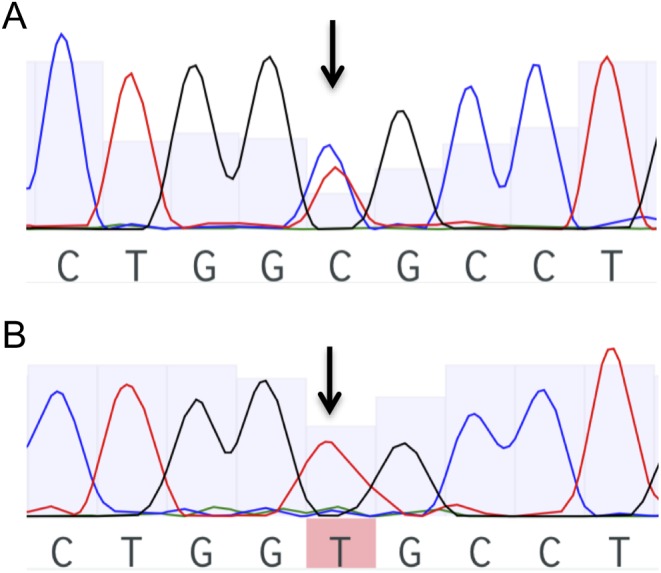
Sequencing electropherograms showing *AIPmut* c.896C > T (A299V) in exon 6. Black arrows show the position of the nucleotide change. (A) Blood leukocyte genomic DNA from acromegaly patient. (B) Tumor genomic DNA from acromegaly patient with loss of heterozygosity (LOH).

Of the 12 genomic DNA tumor samples from our cohort, 9 were from patients who had no mutation on *AIP* sequencing of the peripheral blood DNA (6 somatotropinomas, 2 prolactinomas and 1 Cushing’s disease). These samples did not show somatic *AIP* mutations. Among the 3 patients that have shown an *AIPmut* on the peripheral blood leukocyte DNA analysis, LOH was investigated through *AIP* sequencing. LOH was found in the tumor sample of the acromegaly patient harboring the A299V (c.896C > T) VUS, with loss of the wild-type allele (Fig. 3A and B). No LOH was identified in the tumor sample from the patient with Cushing’s disease harboring the A299V VUS and the patient with acromegaly harboring the c.*14C > A *AIPmut*.

### *AIPmut* in pediatric patients with pituitary adenomas

Considering only pediatric patients, *AIPmut* were identified in 2 of 15 patients (13.3%) diagnosed until 18 years of age. The pathogenic *AIPmut* R304Q was found in one patient with prolactinoma and the *AIP* VUS A299V was found in one patient with Cushing’s disease. Dosage analysis by MLPA was possible from 12 of the 13 pediatric patients without *AIPmut* and did not reveal any large deletions.

## Discussion

Our finding of *AIPmut* in 8.3% of our cohort and in 13.3% among pediatric patients is consistent with the findings of previous studies in other populations (13, 14, 16, 24). Moreover, our study identified two *AIPmut* exclusively found in Brazilian patients and also shows, for the first time, LOH in the tumor DNA from an acromegaly patient harboring the A299V *AIPmut*. Although the study has included all types of pituitary adenomas matching the inclusion criteria, the acromegaly group is overrepresented, probably because we are a state referral center for the treatment of acromegaly, and the NFPA group has a limited number of patients; therefore, we cannot draw a definitive conclusion on the prevalence of *AIP* mutations in this type of adenoma.

Although *AIPmut* frequency in Brazil has shown to be similar to other populations, there are some interesting aspects of our study. Among the eight different *AIPmut* found, two out of three pathogenic mutations were never described in other populations. The first one is the nonsense *AIPmut* Y268*, which has been found in a Brazilian family with acromegaly (31), in a Brazilian patient with familial macroprolactinoma diagnosed in his twenties (18) and in the patient with gigantism presented here and elsewhere (14). This mutation results in a premature TAA-stop signal at codon 268 (31) (Table 2), and a missense mutation at this residue has previously been reported (*AIPmut* Y268C) (18). Another patient from our center who was diagnosed with acromegaly at 49 years, tested positive for the same *AIPmut* in another study (32). However, no family relationship between them was found, but a founder effect cannot be excluded. Family members of both patients were screened for the *AIPmut* Y268* and carriers’ relatives were found. The second one is the novel germline frameshift *AIPmut* K273Rfs*30 predicted to lead to a truncated protein (Table 2). This mutation was only described in our patient with gigantism (current study and (14)). Both mutations are located on exon 6, which is the most affected exon of the *AIP* gene.

The third pathogenic mutation found is the missense mutation R304Q (c.911G > A) (Table 2), first described by Georgitsi and coworkers (33) in a seemingly sporadic case of Cushing’s disease. Our study identified this mutation in an 18-year-old patient with a giant prolactinoma resistant to CAB treatment, with his unaffected father and uncle carrying the same mutation. The pathogenic role of R304Q has been strongly supported by clinical data, since it has been identified in several independent FIPA families as well as in sporadic patients including cases of acromegaly, Cushing’s disease and prolactinoma (9, 11, 12, 13, 16, 24, 25, 33). The c.911G > A is part of a CpG island mutational hotspot (34) and the missense mutation could possibly affect the interaction of AIP with AhR (33), but functional studies did not show significant reduction in β-galactosidase activity for the R304Q AIP mutant (11, 35) (Table 2). Moreover, the MAF of *AIPmut* R304Q, provided by ExAC, is very high when compared to other pathogenic or VUS *AIPmut* (Table 2). Therefore, the higher MAF together with discrepancies between experimental conditions and clinical data could lead to a review of the classification of the *AIPmut* R304Q to a likely pathogenic mutation or even to a VUS.

The *AIP* VUS A299V (Table 2) has been found in patients with acromegaly, prolactinoma and NFPA, both in sporadic and familial cohorts (11, 14, 15, 20, 33). None of these studies showed tumor LOH related to this VUS, but there are also no data of this VUS being found in any of the large general population databases. We identified the *AIP* VUS A299V, in a young patient with Cushing’s disease and in a patient with acromegaly resistant to treatment with SA. Both tumor samples were available, and LOH was found in the somatotropinoma, with retention of the mutated allele, which is in accordance with the Knudson’s two-hit hypothesis (36). Therefore, this is the first time that the LOH of the *AIP* VUS A299V is found in a somatotropinoma supporting the possibility that it might play a role in pituitary adenoma pathogenesis. In contrast, the corticotropinoma did not show LOH. This may be explained by contamination of the tumor sample with normal pituitary tissue, especially because it was taken from a second surgery of this originally 10 mm adenoma. Another possibility is that the Cushing’s disease patient may have a different (i.e. not loss of 11q13 chromosomal material) second hit for the development of the pituitary tumor, such as downregulation of gene expression through promoter methylation (37) or via microRNAs (38).

One of our important findings is that we have not identified somatic mutations in the tumor samples studied. This is in accordance with previous data in the literature (9, 26, 33, 39) and with the Catalogue Of Somatic Mutations In Cancer (http://cancer.sanger.ac.uk) suggesting that somatic *AIPmut* does not seem to contribute in the pathogenesis of the SPA.

The likely benign *AIPmut* R16H (Table 2), first described by Daly and coworkers (40) in a FIPA family, was identified in an acromegaly patient diagnosed at the age of 33 years with a macroadenoma. At first, the *AIPmut* R16H was considered a VUS, but although this mutation has been found in familial and sporadic patients, no LOH was identified in tumor samples, and it has been found in some control subjects, besides it has a high MAF (33, 40, 41, 42). Therefore, it is questioned whether the R16H is pathogenic, and it is hypothesized that it is a rare polymorphism (33, 42, 43).

Regarding the *AIPmut* R128C (c.382C > T), we found it in two acromegaly patients, and it has been previously described in two prolactinoma patients (15). *In silico* analyses predict both a benign and a deleterious mutation (Table 2), and there is lack of functional studies, restricting the conclusions about its pathogenicity. Therefore, we classified it as a VUS. Moreover, another mutation at this site (R128H) has been described in an acromegaly patient (44), suggesting that there might be a pathogenic role for the *AIPmut* R128C. The last two *AIPmut* (c.*14C > A and c.*64G > A) are located at the 3′ UTR; therefore, there is no amino acid change in AIP structure (Table 2). However, the 3′ UTR of *AIP* is a known target for microRNAs (miRNAs), which are small noncoding RNAs that inhibit posttranscriptional expression of target mRNAs by binding to target sequences (45). Therefore, changes in this area could change the affinity of a specific miRNAs to its target. The two most well-studied miRNAs that bind to the *AIP* 3′ UTR are the miR-34a and the miR-107 (14, 32, 45), but their binding sites do not overlap with these two new variants. The c.*64G > A has a high MAF and is classified as likely benign at dbSNP website (Table 2). The c.*14C > A, on the other hand, has a low MAF provided by ExAC (Table 2) and was detected in two patients of our cohort, although no LOH have been found in the tumor sample of one of the patients harboring this *AIPmut*. Therefore, due to these conflicting data, we classified the *AIPmut* c.*14C > A as a VUS.

Large genomic deletions of the *AIP* gene in mutation-negative patients can be detected by MLPA. The majority of studies that have used MLPA did not find large deletions (12, 13, 16, 19, 24, 46, 47, 48, 49, 50). The finding of deletions in blood DNA was restricted to 4 studies, 2 of them including only FIPA patients (11, 51), and other 2 studies, one including only GH-secreting adenomas, both sporadic or FIPA, that found deletions in 2 giant patients, one of them with FIPA (25), and the other one including all types of pituitary adenomas both sporadic or FIPA, that found deletion in 1 giant patient (14). In our study, MLPA analysis was restricted to pediatric and acromegaly patients with quality DNA available, with no detection of large deletions. Our finding is in agreement with previous studies of SPA (12, 13, 16, 24, 46, 47). Therefore, MLPA analysis may be restricted to FIPA cases, for sporadic pituitary adenoma patients diagnosed in childhood or adolescence, or even in patients with a phenotype highly suggestive of *AIPmut*, that were tested negative for *AIPmut* in sequencing analyses.

In conclusion, our *AIPmut* screening performed for the first time in a Brazilian population corroborates the low frequency of germline *AIPmut* in SPA, as previously reported in other ethnic populations. Moreover, we found two *AIPmut* that are only described in Brazilian patients. We also show that special populations like patients with gigantism and patients diagnosed in childhood present a higher prevalence of *AIPmut*, and therefore, should be considered for screening. This allows early identification of affected carriers, when the proband is identified. Finally, we described for the first time the presence of LOH in a somatotropinoma from an acromegaly patient harboring an A299V *AIPmut*, previously classified as VUS. Finally, the diversity of *AIPmut* found among all the studies points to the need for more functional studies for a better understanding of the role of AIP in the pituitary tumorigenesis.

## Declaration of interest

M R G has received consulting fees from Novartis, Ipsen and Ionis, speaker fees from Novartis, Pfizer and Ipsen and research grants from Novartis, Ipsen and Pfizer. P B A is employed by Diagnósticos da América SA, but the company had no interference in the development of the study, and this affiliation does not alter policies on sharing data and materials.

## Funding

The laboratory work was funded by a grant from Fundação de Amparo a Pesquisa do Estado do Rio de Janeiro – FAPERJ E-26/010.001967/2014 (to M R G) and from unrestricted research grants from Novartis and Ipsen (to M R G). The funder Diagnósticos da América SA provided support in the form of salaries for author P B A, but did not have any additional role in the study design, data collection and analysis, decision to publish or preparation of the manuscript.
